# Application of serum Raman spectroscopy combined with classification model for rapid breast cancer screening

**DOI:** 10.3389/fonc.2023.1258436

**Published:** 2023-10-26

**Authors:** Runrui Lin, Bowen Peng, Lintao Li, Xiaoliang He, Huan Yan, Chao Tian, Huaichao Luo, Gang Yin

**Affiliations:** ^1^ School of Medicine, University of Electronic Science and Technology of China, Chengdu, China; ^2^ School of Electronic Science and Engineering, Nanjing University, Nanjing, China; ^3^ Radiation Oncology Key Laboratory of Sichuan Province, Sichuan Cancer Hospital & Institute, Affiliated Cancer Hospital of University of Electronic Science and Technology of China, Chengdu, China; ^4^ School of Clinical Medicine, Southwest Medical University, Luzhou, China

**Keywords:** breast cancer, Raman spectroscopy, machine learning, classification, screening

## Abstract

**Introduction:**

This study aimed to evaluate the feasibility of using general Raman spectroscopy as a method to screen for breast cancer. The objective was to develop a machine learning model that utilizes Raman spectroscopy to detect serum samples from breast cancer patients, benign cases, and healthy subjects, with puncture biopsy as the gold standard for comparison. The goal was to explore the value of Raman spectroscopy in the differential diagnosis of breast cancer, benign lesions, and healthy individuals.

**Methods:**

In this study, blood serum samples were collected from a total of 333 participants. Among them, there were 129 cases of tumors (pathologically diagnosed as breast cancer and labeled as cancer), 91 cases of benign lesions (pathologically diagnosed as benign and labeled as benign), and 113 cases of healthy controls (labeled as normal). Raman spectra of the serum samples from each group were collected. To classify the normal, benign, and cancer sample groups, principal component analysis (PCA) combined with support vector machine (SVM) was used. The SVM model was evaluated using a cross-validation method.

**Results:**

The results of the study revealed significant differences in the mean Raman spectra of the serum samples between the normal and tumor/benign groups. Although the mean Raman spectra showed slight variations between the cancer and benign groups, the SVM model achieved a remarkable prediction accuracy of up to 98% for classifying cancer, benign, and normal groups.

**Discussion:**

In conclusion, this exploratory study has demonstrated the tremendous potential of general Raman spectroscopy as a clinical adjunctive diagnostic and rapid screening tool for breast cancer.

## 1 Introduction

According to the World Health Organization’s International Agency for Research on Cancer, breast cancer surpassed lung cancer as the leading type of cancer worldwide in 2020. In China, due to lifestyle changes, the incidence of new cases of breast cancer has ranked first among all female malignancies. Notably, both the incidence and death rates of breast cancer are steadily increasing year by year, and the age of onset is progressively becoming younger (1–4). Breast cancer represents a prevalent malignant tumor that significantly impacts women’s survival and quality of life (5). Detecting the disease at an early stage and initiating timely treatment are vital strategies for controlling disease progression, enhancing treatment effectiveness, reducing mortality rates, and ultimately improving patient prognosis (6).

Breast cancer screening is widely recognized as a crucial preventive measure that effectively facilitates the early diagnosis, treatment, and cure of breast cancer patients (7). The appropriate utilization of effective screening techniques has the potential to enhance survival rates and reduce mortality. Currently, clinical breast cancer screening predominantly relies on various imaging methods such as breast ultrasound, mammography, and magnetic resonance imaging (MRI). Among these techniques, mammography is primarily recommended in the guidelines of European and American countries for breast cancer screening (8). However, the sensitivity of mammography decreases significantly from 98% in fatty breasts to 48% in dense breasts. Considering that Chinese women tend to have a higher proportion of dense breasts and an earlier age of onset compared to Western women, the sensitivity of X-ray screening can be compromised, and there may even be radiation risks. Consequently, this screening approach may not be suitable for the Chinese population (9). MRI is another commonly employed breast examination technique with a sensitivity ranging from 94% to 100%. However, it can lead to a high rate of over-treatment due to its relatively low specificity (10). Additionally, MRI scans are more expensive compared to other methods. Ultrasound is widely utilized for breast cancer screening and early diagnosis, but it suffers from low resolution and a higher margin of error. Furthermore, breast cancer is often insidious in its early stages, displaying no obvious symptoms. Imaging diagnostic images exhibit a high degree of similarity between cancerous and non-cancerous cases. As a result, imaging is typically used as an adjunct method, providing initial identification of tumor shape and type but unable to accurately discern the tumor’s nature (11–14). Consequently, histopathological examination remains the gold standard for diagnosing breast cancer. However, this method involves invasive procedures, is time-consuming, and lacks diagnostic timeliness, thereby limiting its widespread application in screening (15–19). In routine breast examinations, imaging and physical examination are typically conducted, and malignant suspicious masses require puncture biopsy and histopathology examination. Approximately 70% to 90% of patients are diagnosed as benign, leading to unnecessary trauma, mental stress, and financial burdens for patients (20–22). Therefore, there is an urgent clinical need for an objective, rapid, convenient, and sensitive screening method for early detection of breast cancer.

Raman spectroscopy is a spectroscopic technique that leverages the Raman scattering effect to capture and transmit intrinsic information about the chemical structures of diverse bio-molecules. It provides unique “molecular fingerprint” features for label-free, labeled, and quantitative analysis of cells, tissues, body fluids, and other biological samples (23, 24). Raman spectroscopy offers several advantages, including non-invasiveness, no requirement for sample preparation, non-contact measurements, preservation of sample structure, rapid analysis, ease of operation, and high resolution, etc. These attributes have demonstrated exceptional value in various research areas, such as tumor diagnosis, disease prediction, and understanding pathogenesis. In particular, serum, which primarily consists of water, carbohydrates, proteins, phospholipids, and polysaccharides, exhibits distinct Raman fingerprint spectra, with Raman signals of these components stronger than that of water. The concentrations of these biological substances are closely related to the secretion and degradation of cells or tissues. Malignant transformation brings about morphological and functional changes accompanied by significant biochemical alterations that inevitably manifest in alterations in the composition, type, and content of various substances in the serum (25). Raman-based methods can effectively detect and quantify the molecular signature of these changes, providing valuable references for cancer diagnosis. Moreover, serum is easily accessible, further emphasizing the significance of serum Raman spectroscopy-based analysis and research in the field of biomedicine. For instance, previous study has demonstrated the potential of Raman spectroscopy based on serum as a safe and effective screening technique for COVID-19 (26). Surface-enhanced Raman spectroscopy (SERS) has also been employed to detect serum samples from patients with lung nodules and healthy individuals, revealing the great potential of nanoparticle-based SERS combined with SVM as a clinical auxiliary diagnostic and screening tool for lung adenocarcinoma nodules (27). Additionally, Pichardo-Molina et al. (25) conducted Raman spectroscopy analysis on serum samples from 11 breast cancer patients and 12 healthy individuals, utilizing machine learning to identify differences between the control and experimental groups. This research indicated that Raman spectroscopy can assist in the detection of breast cancer compared to healthy individuals, and that serum-based Raman spectroscopy holds promise for breast cancer diagnosis. Zeng et al.’ research (28) introduced a rapid diagnostic method based on serum Raman spectroscopy and convolutional neural networks for screening triple-negative breast cancer, HER2-positive breast cancer, and a healthy control group. The study achieved favorable diagnostic outcomes. Considering the limitations of traditional diagnostic methods, the development of a non-invasive, fast, cost-effective, and user-friendly breast cancer adjunct diagnostic technology holds significant value. Raman spectroscopy is a powerful analytical technique capable of measuring the content of biomolecules in serum samples. When combined with deep learning algorithms, it can establish classification models based on Raman spectroscopy, enabling quantitative and objective diagnosis of patients. This study also highlights the potential for a rapid, cost-effective breast cancer screening method using serum Raman spectroscopy and deep learning algorithms.

In the past few decades, various Raman spectroscopy applications for breast cancer research have been conducted worldwide, providing data support for the development of breast cancer screening programs. However, most current research has primarily focused on the detection of breast cancer cells, tissues, and tumor markers. With the continuous advancement of Raman spectroscopy technology, Surface-Enhanced Raman Spectroscopy (SERS) has emerged. SERS effectively enhances the weak Raman spectral signals of biomacromolecules by utilizing noble metals (such as gold, silver, copper) or composite materials containing noble metals as substrates. Consequently, SERS technology has rapidly developed and garnered extensive attention in the field of biomedical research. Currently, most studies are exploring the significant value of SERS in breast cancer screening, diagnosis, and postoperative assessment. For example, Enrique and his team’s study (29) successfully achieved breast cancer detection using surface-enhanced Raman spectroscopy (SERS) on serum samples. By analyzing characteristic peaks in the Raman spectra, they could differentiate normal samples from cancer samples with high sensitivity and specificity. This non-invasive diagnostic tool has the potential to complement current detection techniques, requiring minimal sample preparation and providing objective, specific, and rapid results. Cervo and colleagues’ research (30) explored the potential of surface-enhanced Raman scattering (SERS) analysis of serum as a candidate method for detecting early and locally advanced breast cancer. The study utilized serum samples from three groups of participants and established predictive models through principal component analysis (PCA) and linear discriminant analysis (LDA). The performance of these models was assessed through cross-validation. The research findings indicate that SERS spectroscopy combined with multivariate data analysis can differentiate between healthy individuals and breast cancer patients, even distinguishing between different clinical stages of breast cancer. However, there are limited articles that apply regular Raman spectroscopy to serum samples from breast cancer patients. While many studies have demonstrated the diagnostic potential of serum SERS for breast cancer, the preparation and application of the surface-enhanced substrates, such as gold or silver nanoparticles, associated with SERS technology, can be complex and may lack stability, making it less feasible for implementation, particularly in grassroots medical facilities. In order to explore a more convenient and readily accessible diagnostic technology, this study aims to investigate the feasibility of applying regular Raman spectroscopy to breast cancer screening.

Building upon the aforementioned background, we wonder to investigate the feasibility of utilizing general Raman spectroscopy as a screening tool for breast cancer, offering a more convenient and accessible diagnostic technique. In this study, we aim to test the Raman spectra of serum samples obtained from breast cancer patients, benign cases, and healthy individuals. By combining these spectra with machine learning algorithms, we intend to employ mathematical methods to analyze the results. This approach enables us to transform the challenge of material recognition of Raman spectroscopy into a classification problem of machine learning. This study is a retrospective study with pathological diagnosis as the gold standard to label serum. SVM was used to establish a classification model for analyzing the benignity and malignancy of breast neoplasms. Our study endeavors to explore the significance of utilizing Raman spectroscopy for early breast cancer screening, with the intention of establishing a foundation for selecting appropriate diagnostic modalities and equipping clinicians with valuable auxiliary tools for surgical interventions and accurate diagnoses. By exploring the value of early breast cancer screening, we seek to contribute to the advancement of diagnostic practices and improve patient outcomes. This research has the potential to enhance the overall management of breast cancer, enabling timely interventions and personalized treatment strategies based on early detection.

## 2 Materials and methods

### 2.1 Research object

This study, with the approval of the Ethics Committee for Medical Research and New Medical Technology of Sichuan Cancer Hospital (IRB approval number SCCHEC-02-2022-140), obtained informed consent from all subjects. The serum samples used in this study were collected at Sichuan Cancer Hospital in China, adhering to specific inclusion and exclusion criteria.

(1) Controls group: ① All healthy subjects in the control group were female. ② No significant abnormalities in blood routine, hepatic function, and renal function. ③ No history of malignant tumors. ④ No organ dysfunction of the heart, liver, or kidneys. ⑤ Healthy subjects who had not undergone radiotherapy, chemotherapy, or immunotherapy were included.
(2) Benign breast lesions group: ① All patients in this group were female. ② Pathological diagnoses confirmed the presence of benign conditions such as fibroadenoma, adenopathy, intraductal papilloma, mammary hyperplasia, among others. ③ There was no history of any other malignant tumors among the patients. ④ Participants did not exhibit any dysfunction in their heart, liver, kidneys, or any other organs. ⑤ No surgery, radiotherapy, chemotherapy, or other treatment was administered prior to the collection of serum samples.
(3) Breast malignant tumor group: ① All patients in this group were female. ② Pathological diagnoses confirmed the presence of malignant breast tumors. ③ Participants did not have a history of any other malignant tumors. ④ Individuals did not present any dysfunction in their heart, liver, or kidneys. ⑤ No surgery, radiotherapy, chemotherapy, or immunotherapy had been administered prior to sample collection.

The control group consisted of 113 healthy individuals, with an average age of 46.40 years. The cancer group included 129 patients, with an average age of 46.97 years. Additionally, the benign group comprised 91 patients, with a mean age of 46.43 years. Detailed information regarding age, clinical stage, disease location, and other relevant characteristics of all individuals can be found in **Table 1**.

**Table 1:** Clinical characteristics of individuals under investigation.

	Cancer	Benign	Normal	p value
Total	129	91	113	
Age, y	46.97 ± 6.79 (28~57)	46.43 ± 8.94 (28~69)	46.40 ± 7.15 (29~64)	0.418
Location				
Left	65	49		
Right	64	42		
Clinical stage				
Stage I	104			
Stage II	25			
tumor size	1.08 ± 0.60	1.07 ± 0.57		0.963

### 2.2 Test method

Introduction of the instrument: The instrument used in this study is a medical Raman spectrometer. Its model is RTS Endoscopy (Zolix, Beijing, China). This system comprises several key components, including a laser, Raman probe, deep-cooled CCD camera, volume phase holography (VPH) spectrometer, among others. The Raman probe has a spectrometer end, a sample end, and a laser end. The spectrometer end of the system is equipped with an SMA fiber adapter and sequential double-edge filters. These filters effectively block any back-scattered Rayleigh signals, allowing only Raman signals to pass through.The sample end has been customized to securely hold the cuvette, which acts as the container for the serum samples being tested. On the other hand, the laser end features an SMA fiber adapter and collimating lens, enabling the generation of a well-collimated 785nm laser. Moreover, an internal laser line filter is employed within the system to ensure a clean laser profile. In the instrument, the spectrometer was equipped with a thermoelectrically cooled CCD camera, and the Raman signal was focused into an optical fiber through a lens and then the signal was directed into the spectrometer through that fiber. For this study, a single-mode semiconductor laser with a wavelength of 785 nm was employed for Raman excitation. However, it should be noted that the laser power applied to the test samples during experimentation was adjusted to approximately 70 mW to maintain optimal conditions and minimize any potential impact on the samples.

Collection of serum: For each subject, a total of 2 mL of fasting venous blood was collected between 6-7 am on the following day, following an 8-hour fasting period. It is important to note that no anticoagulants were added to these blood samples. Subsequently, the collected blood samples were subjected to centrifugation at 4000 R/min for 10 minutes using a centrifuge. This process facilitated the separation of the upper serum layer, which was then extracted as the sample for analysis. Once collected, all serum samples obtained from the participants were promptly stored in a refrigerator at a temperature of -80 °C to ensure preservation until further testing. This storage condition was chosen to maintain the integrity and stability of the samples for subsequent analyses.

Experimental operation steps: To ensure consistency and accuracy in our experimental procedures, we followed the following steps. Firstly, we stored the serum samples obtained from the inspection section of the Sichuan Cancer Hospital in a refrigerator at -80°C until further testing. The time taken from thawing to testing was kept consistent for each serum sample. Secondly, according to this research protocol, before we started the experiment (i.e. before sampling the serum samples), we performed spectral calibration of the spectroscopic instrument using a neon lamp. Next, we loaded a specific quantity of anhydrous ethanol into the cuvette and used an exposure time of 3 seconds to measure the spectrum of alcohol. Thus performed wave number calibration. This process facilitated wavelength calibration, which was essential for subsequent analysis. Thirdly, after completing the calibration steps, we proceeded by adding each serum sample to the cuvette individually. We then measured the Raman spectra of the serum using the same integration parameters. It was crucial to maintain a light-proof environment throughout the experiment, and all the operations were performed by the same person to ensure consistency. We placed the cuvette in a specific slot of the Raman spectrometer to ensure that the laser path passes through the wall of the tube at a certain angle. Lastly, during spectral acquisition, we collected spectra in the range of 200 to 2000 cm^-1^. After cosmic-ray removal from the spectral data, each serum sample underwent 10 scans conducted by the same experimenter. Multiple spectra were collected for each sample to ensure the accurate characterization of the heterogeneous composition of the sample. Raman data were recorded 10 times for each serum sample and the mean spectrum for each sample was taken for further analysis.

### 2.3 Data processing and analysis

The acquired raw Raman spectra from the serum samples exhibited prominent fluorescence backgrounds and noise, necessitating preprocessing of the spectral data before conducting analysis. Our objective was to preprocess the 3330 spectra obtained from 333 serum samples to identify molecular bands and functional groups. All the spectral lines were intercepted in the range of 600 cm^-1^~1800 cm^-1^. Additionally, due to potential background shifts caused by instrumentation, we performed noise reduction and baseline removal on each raw spectrum (31). To extract the pure Raman signals, a Vancouver Raman algorithm based on a seventh-order polynomial was employed to fit all serum auto-fluorescence backgrounds, this polynomial was then subtracted to correct the baseline. Subsequently, each background-subtracted Raman spectrum was normalized by the integrated area under the curve. Thus, the influence of spectral intensity variability generated by possible laser power fluctuations could be reduced, and the spectral shapes and Raman peak intensities could be compared between the different groups of serum samples (32). Simultaneously, we also conducted an average processing of the Raman spectroscopic data from the serum samples. Each serum sample was recorded ten times, and the average value was taken as the data representing that particular serum sample for subsequent analysis and modeling. (We used the average of the spectra for each serum sample as the datapoint for the SVM analysis.) Following these preprocessing steps, the spectral data were transformed into normalized data for training purposes. Principal Component Analysis (PCA) was then applied to the training data to extract spectral features. We selected the principal components that retained 99% of the information as the features for further analysis. PCA was applied to extract spectral features and thus classify individual spectra based on their Raman spectral fingerprints. PCA used a singular value decomposition method to decompose independent variations as principal components (PCs), where the contribution of each PC was referred to as its score. Then a linear transformation was applied to omit less important variables and displayed the features of the original data for dimensionality reduction purposes. PCA is a widely used classical tool for feature extraction in multivariate statistical analysis. Finally, the preprocessed data described above was utilized for classification purposes, enabling the identification and differentiation of serum samples based on their Raman spectral fingerprints.

### 2.4 Model establishment and verification

This article focuses on the combination of Raman spectroscopy with machine learning algorithms for data classification. Machine learning algorithms provide an effective means of analyzing Raman spectral data, enabling the extraction of useful features from each dataset and subsequent classification based on these features. Support vector machine (SVM) is renowned for its capability to effectively handle complex datasets and high-dimensional feature spaces. In this study, our Raman spectroscopic data inherently exhibits non-linearity, and SVM is well-suited to capture complex, non-linear relationships within the data. This characteristic of SVM allows it to potentially model the underlying structure of the data more effectively than linear methods. The choice of classification algorithm can also be influenced by specific research objectives and expected outcomes. Our primary research goal was to accurately classify serum samples into different categories (normal, benign, malignant) based on their Raman spectral fingerprints. While linear methods like PLS-DA can be effective, our specific aim was to capture potential non-linear patterns in the data. SVM’s capacity to model non-linear relationships aligned better with our research objectives. Furthermore, previous research (33) has demonstrated the significant potential of label-free serum surface-enhanced Raman analysis combined with support vector machine diagnostic algorithms in non-invasive prostate cancer screening. Their study found that SVM’s diagnostic performance surpasses that of linear algorithms like PCA-LDA. Because the support vector machine (SVM) is a relatively young multivariate technique, it is considered superior to traditional linear methods due to its ability to handle binary classification problems with non-linear boundaries by mapping sample datasets into higher-dimensional spaces. We also have previous research experience (27) that demonstrates the effectiveness of SVM in handling similar types of data. SVM has achieved success in prior studies with similar features; hence, in this study, SVM was once again the chosen classification method. Finally, it is worth noting that SVM provides the flexibility to adapt to various datasets by adjusting parameters such as the kernel function and regularization. This adaptability can help mitigate the risk of overfitting. For the above reasons, in our study, SVM stands out as a highly effective classifier among various existing algorithms. Not only does SVM classify the data, but it also optimizes the decision boundary by maximizing the margin between data clusters. It is worth noting that SVM is a linear binary classifier (34). However, in the case of multivariate datasets, we employed a one-vs-all multi-class implementation to transform the binary classifier into a multiple-class discrimination model (35, 36). Furthermore, we utilized LIBSVM within the MATLAB environment to tri-classify the testing data into three categories: normal, benign, and malignant. LIBSVM is a comprehensive software tool designed for support vector classification, regression, and estimation of distribution. It supports various SVM formulations, efficient multi-class classification, cross-validation of model selection, weighted SVM for unbalanced data, and automatic model selection to generate cross-validation accuracy profiles, among other features (37).

Upon completing the aforementioned operations, we ventured even further, implementing a dual-layer cross-validation scheme to avoid overestimation and over-fitting, thereby scrutinizing the algorithm’s performance in the classification of Raman spectral data (38). Within this scheme, we partitioned the data into two distinct components: the model building dataset and the independent testing dataset. To construct the SVM model, we randomly allocated 80% of the data for model development. This 80% subset was then divided into two subparts with 5-fold cross-validation. Specifically, 80% of the data served as the training set, while the remaining 20% constituted the validation set. Subsequently, we proceeded to test performance using an entirely independent 20% subset, distinct from both the training and validation sets. We repeated the process 100 times until all samples were tested independently. Each fold was used only once as a completely independent test set. We looped the process 100 times for accurate performance estimation.

**Figure 1:**
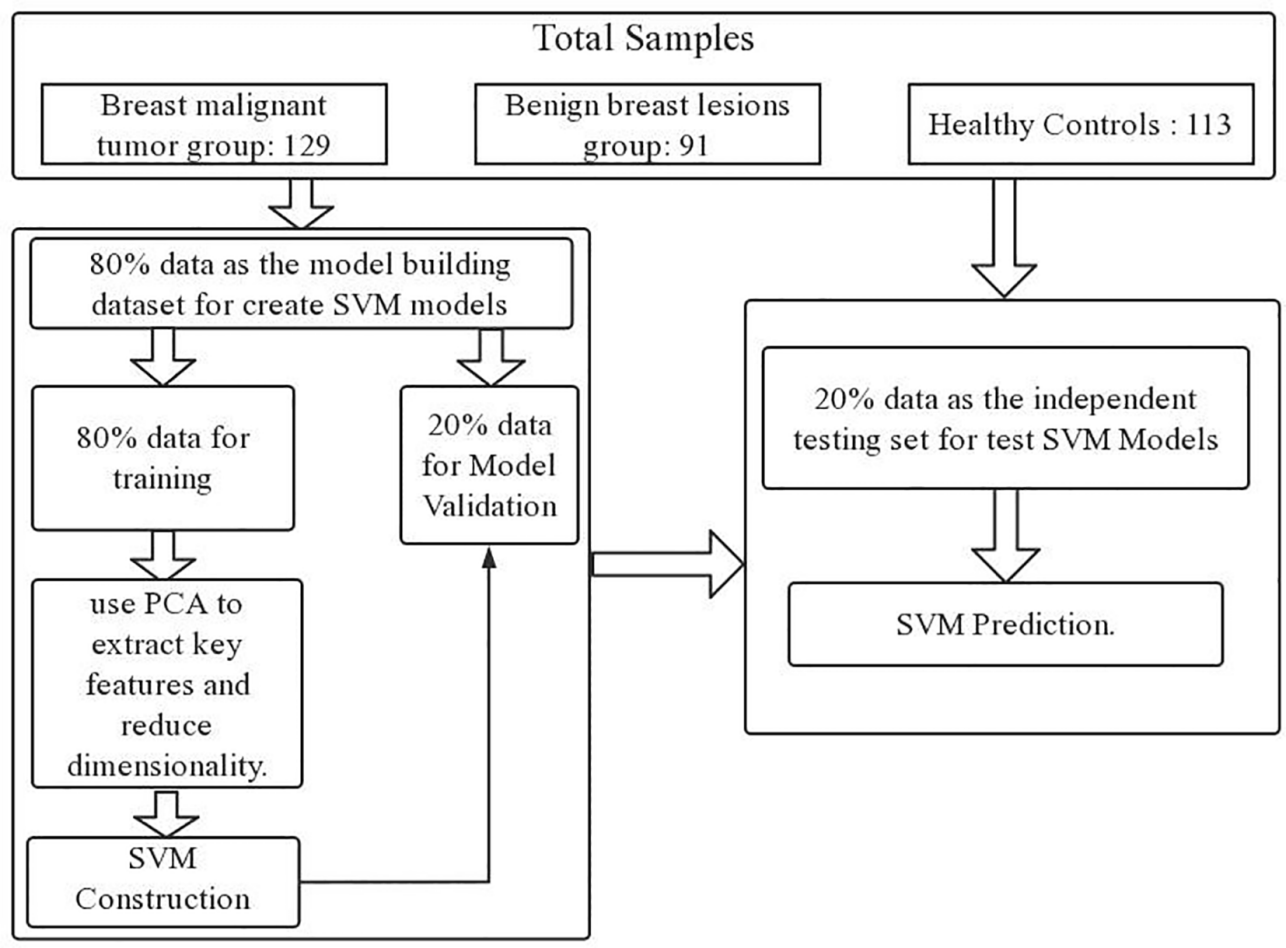
Model establishment and verification.

The flowchart for the development and validation of the SVM prediction model for breast cancer is shown in **Figure 1**.

## 3 Results

### 3.1 Clinical features

In the cancer group, the median age of female patients was 48 years old, with an age range of 28-57 years. In the benign group, the median age of female patients was 46 years old, and their age range was 28 to 69 years. In the normal control group, the median age of these healthy subjects was 47 years old, ranging from 29 to 64 years.

### 3.2 Raman spectroscopy and statistical analysis

**Figure 2:**
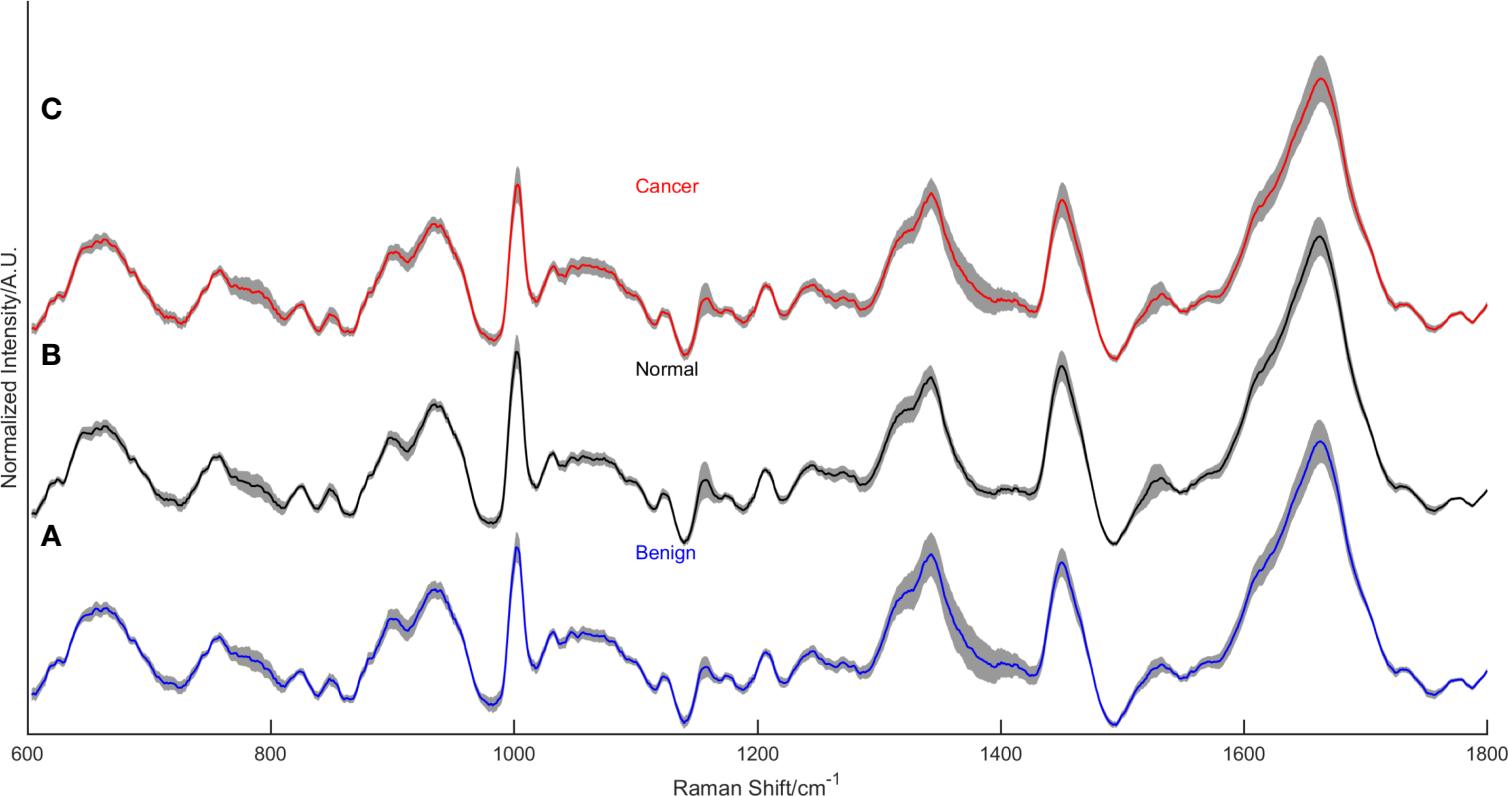
The mean normalized Raman spectra of the benign lesion group **(A)**, the normal control group **(B)**, and the breast cancer group **(C)**.

As we can see in **Figure 2**, there are some differences between the average Raman spectrum of serum from breast cancer patients and serum from healthy individuals. Since both groups had a significant presence of biomacromolecules in their respective serum samples, there were similarities in the vibrational information within the spectra. However, the peak intensities differed due to variations in the content of each biomacromolecule present in the serum. Several representative peaks were identified that showed differences between the experimental group and the control group. Comparative analysis revealed significant differences in spectral peaks at 784cm^-1^, 835cm^-1^, 925cm^-1^, 986cm^-1^, 989cm^-1^, 1002cm^-1^, 1020cm^-1^, 1056cm^-1^, 1114cm^-1^, 1127cm^-1^, 1139cm^-1^, 1285cm^-1^, 1295cm^-1^, 1346cm^-1^, 1367cm^-1^, 1437cm^-1^, 1531cm^-1^ and 1650cm^-1^. **Table 2** provides details on the main differences in spectral variations between breast cancer and control groups, along with the assignment of characteristic peaks (39–51).

**Table 2:** Average position and Assignment of Raman spectroscopy of serum samples.

Peak Position(cm^-1^)	Band Assignment	Tentative Contribution
784 cm^-1^	Phosphate backbone of DNA δ(C_3_CO) deformation	Cytosine L-Histidine Citric acid
835 cm^-1^	para-substituted benzene ring	Tyrosine
925cm^-1^	C(6)-OH	D-Mannose L-Glutamate D-(-)-Fructose
986cm^-1^		Proline
989cm^-1^		Tryptophan
1002cm^-1^	trigonal ring breathing of the benzene ring. N(C–O) stretch	Phenylalanine β-D-glucose N-Acetylglucosamine
1020cm^-1^	indole ring ν(C–O) and ν(C–C) stretches	Tryptophan N-Acetyl-D-glucosamine glucose Glucuronic acid Lactose D-(+)-Galactosamine
1056cm^-1^		L-Glutamate
1114cm^-1^	C-C stretch	breastlipid
1127cm^-1^		Amino acids Fatty acids Saccharides D-fructose-6-phosphate
1139cm^-1^	stretching vibrational ν(C-N)	D-Mannose Amide III
1285cm^-1^	α-helix	Amide III phosphatide
1295cm^-1^	δ(CH_2_) twist vibrations	Fatty acids
1346cm^-1^		Glycine ˛α-D-glucose
1367cm^-1^	CH_3_ indole rings	Tryptophan
1437cm^-1^	CH_2_ scissoring, δ(CH_2_, CH_3_) Bending vibrations	Lipids
1531cm^-1^	ν(C=C) stretching	β-carotene
1650cm^-1^	α-helix	Protein Amide I

**Figure 3:**
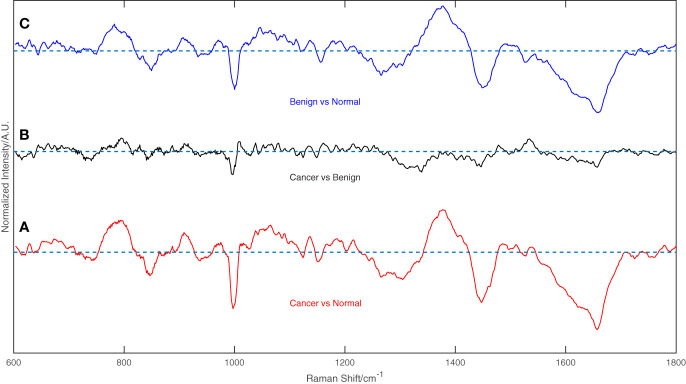
The difference between any two groups of the spectrum, **(A)** Breast cancer vs. Normal groups, **(B)** Breast cancer vs. Benign groups, and **(C)** Benign vs. Normal groups.

**Figure 2** displays the mean normalized spectra of the breast cancer group, the benign lesion group, and the normal control group. The shaded colors in the figure indicate the standard deviation, providing insight into the data variability. In **Figure 3**, the spectra differences between any two groups are shown separately. Observing the figure, it becomes evident that the molecular fingerprints of the spectra of the breast cancer group closely resemble those of the benign lesion group, potentially leading to mislabeling of some samples from these groups by the classifier. However, the figure also highlights a clear distinction between the spectra of the normal group and those of the breast cancer or benign groups.

**Figure 4:**
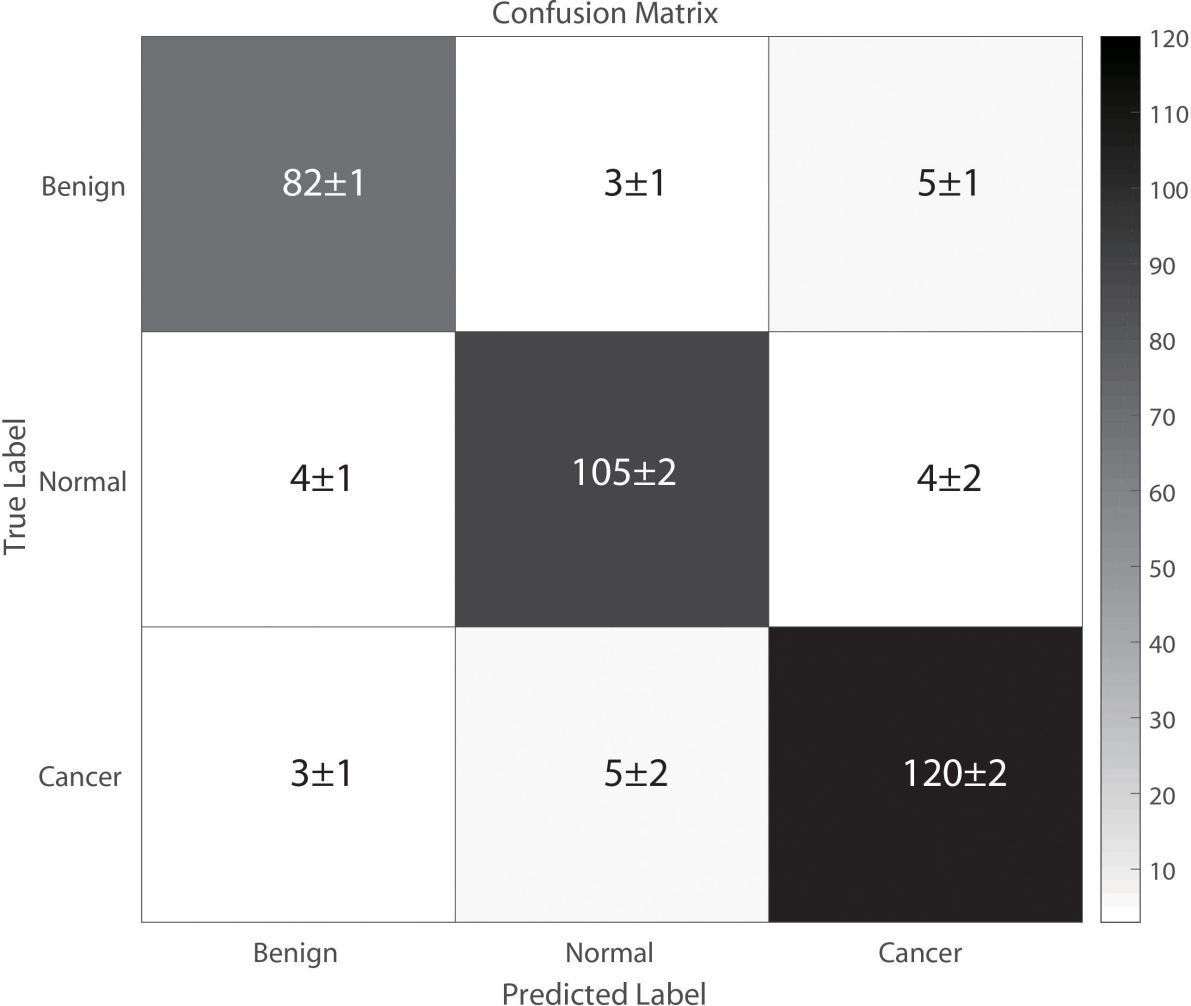
The classification results from our proposed SVM model.

**Figure 5:**
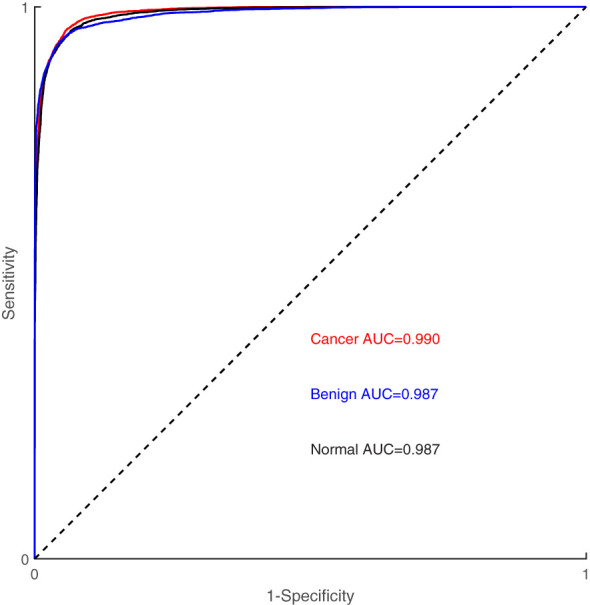
Receiver-operating characteristic (ROC) curve of SVM model.

**Figure 4** illustrates the classification results of breast cancer, benign lesions, and normal individuals using the SVM model proposed in this study, achieving an overall accuracy rate of 98%. The figure shows that out of the 129 samples with malignant breast cancer, only 3 ± 1 samples were incorrectly labeled as benign, and 5 ± 2 samples were incorrectly labeled as normal. Among the 91 benign cases, 8 ± 2 samples were misclassified, with 5 ± 1 cases classified as cancer and 3 ± 1 cases classified as normal. In comparison, 8 ± 3 of the 113 normal control samples were incorrectly classified, with 4 ± 1 samples labeled as benign and 4 ± 2 samples labeled as cancer. The classification of spectra between the cancer group and the benign group exhibited a higher error rate due to the close similarity in molecular fingerprints of the Raman spectra within these two groups. In **Figure 5**, the Receiver Operating Characteristic (ROC) curve of the SVM model proposed in this study is depicted. The figure demonstrates that the area under the curve (AUC) for the breast cancer group, benign lesion group, and normal control group are 0.990, 0.987, and 0.987, respectively. These results indicate that our model has a high ability to discriminate between cancers, making it a potential tool for breast cancer screening.

**Table 3** presents the sensitivity, specificity, and accuracy values for each category. Analyzing these values reveals that our proposed SVM model effectively differentiates between breast cancer, benign lesions, and normal individuals. Notably, our machine learning model exhibits a notable advantage in accurately differentiating normal samples, with a high accuracy rate of up to 0.99. Furthermore, the two-level cross-validation and testing of samples in this study were completely independent of the samples used in the optimization. This ensured a reliable and reproducible method that can be extended to real clinical environments.

**Table 3:** Performance parameters of the SVM.

Class	Performance parameter	Value ± *Std*	95% CI
Cancer	Sensitivity	0.932 ± 0.014	0.922-0.945
	Specificity	0.951 ± 0.011	0.952-0.967
	Accuracy	0.987 ± 0.003	0.986-0.989
Benign	Sensitivity	0.901 ± 0.016	0.901-0.910
	Specificity	0.971 ± 0.007	0.968-0.973
	Accuracy	0.986 ± 0.003	0.985-0.988
Normal	Sensitivity	0.931 ± 0.017	0.926-0.937
	Specificity	0.959 ± 0.010	0.956-0.963
	Accuracy	0.990 ± 0.002	0.989-0.991

## 4 Discussion

Currently, there is a lack of effective preventive measures for breast cancer. Early detection and treatment are crucial in reducing mortality rates and improving prognosis. However, the existing methods for clinical tumor diagnosis, including tumor marker detection, imaging diagnosis, and histopathological diagnosis, have several limitations. Tumor marker detection, for example, is a method of early tumor monitoring based on molecular biology and is susceptible to individual differences and false positives caused by benign diseases (52, 53). While genetic sequencing technology of tumor-associated circulating DNA shows promise for breast cancer detection, it is expensive, has complex procedures, and its clinical utility is uncertain (54). Imaging methods, as mentioned in the introduction, carry certain risks and limitations, including high costs, false positives, over-diagnosis, and radiation exposure. Histopathology, considered the “gold standard” for tumor diagnosis, is a complex discipline heavily reliant on the experience and skills of clinical pathologists (55–57). Moreover, the pathological biopsy is more time-consuming, the timeliness of its diagnosis is poor, and it is an invasive modality (58). In summary, these methods are not suitable for rapid screening of early breast cancer in large-scale populations. Therefore, there is an urgent need for a fast, safe, real-time, non-invasive, label-free, sensitive, accurate, and convenient screening and diagnostic technology for breast cancer.

Raman spectroscopy has gained significant attention in the field of biomedicine due to its high sensitivity, non-destructiveness, and ability to provide fingerprint resolution while having minimal impact on the water environment. It has demonstrated the capability to provide specific molecular characterization of various biological samples and substances, including detecting changes in chemical bonds corresponding to biological macromolecules. Raman spectroscopy can express the metabolic and immunological state in the body by detecting the composition of serum. When an organ in our body becomes cancerous, apoptosis or immune abnormalities can lead to alterations in the serum composition, as well as the structure and quantity of various biomolecules. Raman spectroscopy can detect changes in tumor-related metabolites present in the blood during circulation. These biochemical changes manifest before the appearance of common clinical symptoms observed through medical imaging, offering a unique opportunity to explore subtle molecular-level changes in the serum of patients with early-stage breast cancer. Therefore, Raman spectroscopy holds the potential to be an effective tool for early cancer screening (59). Moreover, Raman spectroscopy screening of serum requires only 2-3 mL of blood, posing no harm to the human body. It can be performed even in grass-roots hospitals without various expensive and complicated medical equipment. As a result, Raman spectroscopy-based early screening is a convenient, easily implementable, and cost-effective technique for breast cancer screening. However, it’s important to note that Raman spectroscopy applied to breast cancer screening does not replace the gold standard, which is pathological diagnosis. Instead, it serves as a primary screening method. Nevertheless, the use of Raman spectroscopy is valuable in achieving cancer screening in more and larger populations, facilitating early detection of breast cancer patients.

In this study, by comparing the differences in the spectra, we could obtain the results of the variation in peak intensity. The differences observed among the three groups were influenced by the content of various biomolecules such as proteins, lipids, sugars, and nucleotides. Interestingly, there were also some similar changes in serum Raman signals between the malignant tumor and benign lesion groups, indicating the presence of shared components in the serum of these patients. Spectroscopic analysis revealed an increase or decrease in the percentage of the total Raman active component of certain bio-molecules in the serum of breast cancer patients compared to healthy subjects. For instance, the peak at 784 cm^-1^ corresponded to cytosine, a primary component of nucleic acids. In the cancer group, the intensity of this Raman peak was higher than in the control group. This may be attributed to the accumulation of nucleic acids and circulating DNA in the blood, resulting from cell necrosis and apoptosis during tumor progression (39). Peaks at 835 cm^-1^, 986 cm^-1^, 989 cm^-1^, and 1002 cm^-1^ represented characteristic peaks of amino acids such as tyrosine, proline, tryptophan, and phenylalanine, respectively. These peaks showed lower intensities in the cancer group compared to the controls. In the context of cancer, the rapid and uncontrolled cell proliferation leads to an increased demand for amino acids involved in DNA and protein synthesis, resulting in reduced serum concentrations of these amino acids (40). Phenylalanine has also been considered as a potential tumor marker (41, 42). Furthermore, the peaks at 1020 cm^-1^ and 1367 cm^-1^ were attributed to tryptophan, while 1056 cm^-1^ was attributed to L-glutamate. These peaks were enhanced in the cancer group, indicating elevated levels of free glutamate and tryptophan in patients, which is consistent with previous findings (43). The 1114 cm^-1^ peak was assigned to the C-C stretch of breast lipid, and its intensity was increased in the cancer group, which aligns with findings from Nargis et al. (44). The peak at 1531 cm^-1^ represented carotenoids, we know that the main carotenoids in human blood are lutein, lycopene, and β-carotene, which have antioxidant effects (45). Related studies have shown that breast cancer patients suffer from oxidative stress behavior and their antioxidant capacity was reduced, resulting in increased depletion of antioxidants in the serum and a decrease in β-carotene levels (46, 47). The peak at 1650 cm^-1^ corresponded to the α-helix of amide I, while the peak at 1285 cm^-1^ belonged to the α-helix of amide III. The relative intensities of these peaks decreased in the cancer group, which indicated that the α-helix of amide had been absorbed during the metabolism process and the spatial structure of the main chain might have been disrupted. This suggested an increase in disordered conformation and a decrease in ordered conformation of proteins. Breast cancer cells require nutrients for growth and consume lipids and proteins to meet their energy needs, leading to reduced levels of these components in the blood (48). In conclusion, the reason for these changes is that breast cancer causes the consumption of substances such as carbohydrates, amino acids, and proteins in the tissues or cells during the cancerous process is different from that of normal people. With tumorigenesis and progression, apoptotic and necrotic cells would release various biochemical components, when blood flowed through tumor tissue, these metabolites such as proteins and nucleic acid fragments would enter the circulation and produce unique small changes in the circulating blood micro-environment. These alterations in relative concentrations of relevant biochemical components are directly reflected in the Raman spectra (49–51). The above analysis shows that there are differences in bio-molecules in the blood of breast cancer patients and healthy controls, and the reason for such differences is related to the biological behavior of breast cancer. And further demonstrating the effectiveness of this serum-based Raman spectroscopy analysis method for diagnostic screening and evaluation of breast cancer. It also provides a theoretical basis for the diagnosis of breast cancer using Raman spectroscopy combined with classification algorithms. But it should be noted that the above-oversimplified peak intensity analysis only used limited Raman peak information. Furthermore, there were significant changes and overlaps in serum Raman spectra between normal subjects and cancer patients. Hence, in this study, we used multivariate statistical analysis to combine the entire spectra and automatically identify the most essential diagnostic features to improve the efficiency and differentiation accuracy of serum analysis.

In the research process, we also conducted a negative control study. We performed classification tests by randomizing the spectral labels (normal, benign, tumor) using the same SVM classifier settings as the correct data labels. The test results showed that the accuracy was approximately 43% with a variation of ±5%. This means that by randomizing the spectral labels in the dataset, we effectively disrupted any meaningful correlation between the spectra and the category labels. Through this method, we were able to demonstrate that when category labels are no longer associated with the actual sample features, the classifier’s performance significantly declines. The noticeable drop in SVM classifier performance after randomizing the data labels indicates that our results are not due to random factors. This further adds a layer of credibility to our research results, confirming that the classifier captures meaningful information rather than arbitrary associations. It ensures that the classification results we observed are meaningful rather than accidental or overfit outcomes.

Li et al. (48) conducted an analysis of serum Raman spectra from 171 invasive ductal carcinoma (IDC) patients and 100 healthy volunteers. They employed serum Raman spectroscopy in conjunction with multiple classification algorithms to develop an auxiliary diagnostic method for early detection of breast cancer. Their results showed the reliability of combining serum Raman spectroscopy with classification models under large sample conditions. Another study by Wang et al. (60) involved collecting Raman spectra from the sera of 241 healthy volunteers, 463 breast cancer patients, and 100 Ductal carcinoma *in situ* (DCIS) patients. Their research explored the feasibility of using Raman spectroscopy in combination with convolutional neural network (CNN) to establish a model capable of classifying these three distinct spectra. The results of their study highlighted the potential utility of CNN as an auxiliary diagnostic tool for breast cancer and DCIS. In alignment with these studies, our own research revealed a clear distinction between normal serum and serum samples obtained from individuals with malignant tumors or benign lesions, underscoring the sensitivity of Raman spectroscopy in this domain. Testing the composition of serum enables the expression of the metabolic and immune status within the body. This is significant since alterations in serum composition can occur due to apoptosis or immune abnormalities, and these changes can reflect the difference between malignant and benign tumors. To enhance classification accuracy and facilitate early detection of breast cancer patients, we conducted statistical analysis employing three groups of variables: the normal control group, benign lesion group, and breast cancer malignancy group. This approach allows for more precise categorization of patients and aids in the identification of breast cancer cases at an earlier stage, potentially reducing excessive medical treatment.

The preliminary data from this study suggests that Raman spectroscopy holds promise as a diagnostic tool for breast cancer, comparable in accuracy to existing clinical diagnostic techniques, while providing surgeons with rapid and objective diagnostic information. However, further exploration and development of Raman spectroscopy in the context of breast cancer are warranted. Several key advantages make it an attractive option for diagnosis. Firstly, it demonstrates good sensitivity and specificity, comparable to current clinical techniques. Secondly, it allows for the collection of spectral information without the need for special staining or preparation, enabling real-time and objective diagnosis. Nevertheless, it is important to acknowledge that tumors are complex systems that exist within intricate physiological contexts. Therefore, relying solely on a single-dimensional view of tumors might be limited. To establish the specificity of Raman spectroscopy for breast cancer, it is necessary to consider potential similarities in serum changes across different types of cancer, as well as the influence of diseases in other organs that may impact serum composition. Additionally, in the future, we need to further integrate clinical data with spectral data to strengthen the correlation between spectral fingerprints and the actual health status of patients. Integrating clinical data not only enhances the specificity of the models but also increases confidence in the accuracy of the ground truth labels, thereby increasing the reliability of the SVM classifier. We will strive to enhance the specificity and reliability of the classification model by incorporating additional clinical data. This will enhance the clinical relevance and applicability of our research findings, making them more robust and reliable for breast cancer diagnosis and differentiation. Furthermore, while this study focused on the metabolic and immune perspectives of serum analysis, there is also a need to explore the classification of breast cancer using imaging techniques. Future research could combine imaging and Raman spectra to establish a more comprehensive diagnostic model for breast cancer, enhancing the reliability of diagnostic results. Additionally, it is worth noting that the data in this study are derived from a single-center, and further evidence should be accumulated through joint multi-center prospective studies to strengthen our findings. Currently, Raman spectroscopy technology is not widely adopted or promoted in clinical practice, and the field is still at the stage of experimental research and remains fragmented in terms of technology, screening protocols, and diagnostic criteria. Larger-scale *in vitro* and *in vivo* studies with standardized processes and protocols are necessary to provide more stable and accurate data, as well as to establish diagnostic databases and criteria. Such efforts will enable a more comprehensive and precise analysis of early breast cancer information. Based on existing literature, it is evident that various sample types, Raman devices, and diagnostic algorithms can be utilized in the study of Raman spectroscopy technology for breast cancer screening. However, standardized processes and protocols for diagnosis using Raman spectroscopy have yet to be established, presenting a challenge in achieving uniformity. Nevertheless, as Raman spectroscopy devices and diagnostic algorithms are improved and further refined, taking full advantage of their ease, speed, and nondestructive nature, there is potential for clinical implementation and widespread use in the future. This would contribute to a new type of clinical diagnostic technique for breast cancer, ultimately supporting standardized breast cancer screening at the grassroots level in China. The promotion of early detection and treatment of breast cancer, along with improvements in the overall survival rate of patients, holds significant importance for the well-being and health security of the Chinese population.

While our study has demonstrated the promise of Raman spectroscopy for serum-based classification, there is indeed scope for enhancing the depth and breadth of information extraction from serum samples. Specifically, if we aim to extend the applicability of this method to predict metastatic progression and therapeutic outcomes, it is imperative to consider advanced spectroscopic techniques and emerging approaches. One of the key limitations of spontaneous Raman spectroscopy is its inherent sensitivity, which can pose challenges when attempting to detect subtle variations in serum composition, especially in the context of metastatic disease and therapeutic responses. To address this limitation, we should explore the potential of Surface-Enhanced Raman Spectroscopy (SERS) assays. SERS can significantly amplify Raman signals, enabling the detection of trace-level molecules and providing more detailed information about serum components. Incorporating SERS into our spectroscopic approach could potentially unlock the capability to identify specific biomarkers associated with metastatic progression and therapeutic responses. This would represent a valuable step towards a more comprehensive and predictive serum analysis method.Furthermore, considering the complexity of cancer biology and its heterogeneous nature, a multimodal spectroscopic approach is a promising avenue to explore. By integrating various spectroscopic techniques, such as Raman spectroscopy, fluorescence spectroscopy, and others, we can obtain complementary information about serum samples. This holistic approach can enhance our ability to capture a broader spectrum of molecular and biochemical changes associated with cancer progression and treatment outcomes. Additionally, the integration of machine learning algorithms and data fusion techniques can facilitate the interpretation of multimodal spectral data and improve predictive modeling. These advancements hold the promise of extending the reach of our method beyond classification to the prediction of metastatic behavior and therapeutic responses, contributing to more personalized and effective cancer diagnostics and treatment monitoring.

## 5 Conclusion

In this study, we employed general Raman spectroscopy to detect serum as an initial screening method for breast tumors. Our study included a substantial sample size of over 300 participants, ensuring comprehensive data collection. The findings revealed a significant distinction between the serum of healthy individuals and those with benign lesions or malignant tumors. Our proposed SVM model achieved 98% accuracy in predicting the differential diagnosis of malignant tumors, benign lesions, and healthy individuals. These results demonstrate the considerable potential of serum Raman scattering as an adjunctive diagnostic tool for breast cancer. General Raman spectroscopy emerges as a fast, effective, and convenient approach for classifying and screening Breast Neoplasms, offering complementary diagnostic information for early breast cancer screening. It holds important research value and has the potential to become a new screening method in clinical practice for breast cancer screening. Furthermore, the insights gained from this study can serve as a reference for the diagnosis of other malignancies. In conclusion, Raman spectroscopy proves to be a promising diagnostic tool for breast cancer, warranting further exploration and development. Its ability to provide valuable diagnostic information through serum analysis makes it a valuable addition to the field of breast cancer screening.

## Data availability statement

The original contributions presented in the study are included in the article/supplementary material. Further inquiries can be directed to the corresponding author.

## Ethics statement

The studies involving humans were approved by Ethics Committee for Medical Research and New Medical Technology of Sichuan Cancer Hospital. The studies were conducted in accordance with the local legislation and institutional requirements. The participants provided their written informed consent to participate in this study.

## Author contributions

RL: Conceptualization, Formal Analysis, Software, Validation, Data curation, Investigation, Methodology, Resources, Writing – original draft. BP: Conceptualization, Formal Analysis, Investigation, Methodology, Software, Validation, Writing – original draft. LL: Methodology, Validation, Data curation, Resources, Writing – review & editing. XH: Resources, Formal Analysis, Investigation, Software, Writing – original draft. HY: Writing – original draft, Data curation, Methodology, Validation. CT: Methodology, Resources, Writing – review & editing. HL: Resources, Writing – review & editing, Investigation. GY: Writing – review & editing, Conceptualization, Formal Analysis, Funding acquisition, Project administration, Software, Validation.
